# Misdiagnosis of polymyositis in a young female patient with occult limb-girdle muscular dystrophy

**DOI:** 10.1093/rap/rkad061

**Published:** 2023-07-11

**Authors:** Lara Valor-Méndez, Matthias Türk, Georg Schett, Bernhard Manger, Johannes Knitza

**Affiliations:** Department of Internal Medicine 3, Rheumatology and Immunology, Friedrich-Alexander University Erlangen-Nürnberg and Universitätsklinikum Erlangen, Erlangen, Germany; Deutsches Zentrum für Immuntherapie, Friedrich-Alexander University Erlangen-Nürnberg and Universitätsklinikum Erlangen, Erlangen, Germany; Neurology, University Hospital, Erlangen, Germany; Department of Internal Medicine 3, Rheumatology and Immunology, Friedrich-Alexander University Erlangen-Nürnberg and Universitätsklinikum Erlangen, Erlangen, Germany; Deutsches Zentrum für Immuntherapie, Friedrich-Alexander University Erlangen-Nürnberg and Universitätsklinikum Erlangen, Erlangen, Germany; Department of Internal Medicine 3, Rheumatology and Immunology, Friedrich-Alexander University Erlangen-Nürnberg and Universitätsklinikum Erlangen, Erlangen, Germany; Deutsches Zentrum für Immuntherapie, Friedrich-Alexander University Erlangen-Nürnberg and Universitätsklinikum Erlangen, Erlangen, Germany; Department of Internal Medicine 3, Rheumatology and Immunology, Friedrich-Alexander University Erlangen-Nürnberg and Universitätsklinikum Erlangen, Erlangen, Germany; Deutsches Zentrum für Immuntherapie, Friedrich-Alexander University Erlangen-Nürnberg and Universitätsklinikum Erlangen, Erlangen, Germany

Key message

- Treatment-refractory myositis should be re-evaluated for differential diagnoses, including muscular dystrophies.

Dear Editor, A 36-year-old woman presented to our clinic with a 10-year history of polymyositis. At diagnosis, she was a competitive badminton athlete complaining of increased proximal muscle soreness and tiring easily after sports, especially in the lower extremities. Before the first medical work-up, very high creatine kinase (CK) levels (>3000 U/l; reference: <170 U/l) had been discovered incidentally. Myopathological analyses of two subsequent diagnostic muscle biopsies (gastrocnemius and vastus lateralis muscles) showed variation in fibre size and presence of regenerating and necrotic muscle fibres. A finding of scattered lymphocytic infiltrates and Jo-1 and Scl-70 autoantibodies (+) finally lead to a diagnosis of polymyositis. Subsequently, the patient had been treated with AZA, MTX, IVIG, tacrolimus, everolimus, MMF, CSA, tocilizumab, rituximab and prednisone at varying doses, with no significant impact on symptoms.

At the time of medical re-evaluation at our clinic, she reported progressive proximal myalgias, muscle fatigue and muscle weakness, which made climbing stairs challenging, but exertional pain was most impairing. Clinical examination revealed a Manual Muscle Test-8 (MMT8) of 142/150. Comprehensive work-up excluded systemic inflammatory disease and, in particular, pulmonary or cardiac involvement. Consistent with the patient’s medical history, laboratory testing reproduced marked elevation of CK (>8000 U/l) and myoglobin (>700 mg/l; reference: <70 mg/l), in addition to fluctuating weak positive (+) Jo-1 and Scl-70 autoantibodies in an ANA immunoblot. ANA titres remained negative. The EUROLINE Inflammatory Myopathies 16 Ag (IgG) commercial line blot immunoassay (Euroimmun, Lubeck, Germany) returned consistently negative results. No relevant inflammatory parameters or other autoantibodies were found. Despite complete B-cell depletion, a second attempt at therapy with rituximab did not improve any symptoms either. At that time, MRI of the thigh muscles (Fig. 1) showed marked fatty atrophy, in particular of the biceps femoris (long head), semitendinosus and adductor magnus and longus muscles, but no relevant oedema. Therefore, neuromuscular re-evaluation was initiated. Neurological examination revealed mild to moderate proximal tetraparesis, calf hypertrophy and a myopathic pattern, but no spontaneous activity on EMG. There was no family history for neuromuscular disorders.

**Figure 1.**
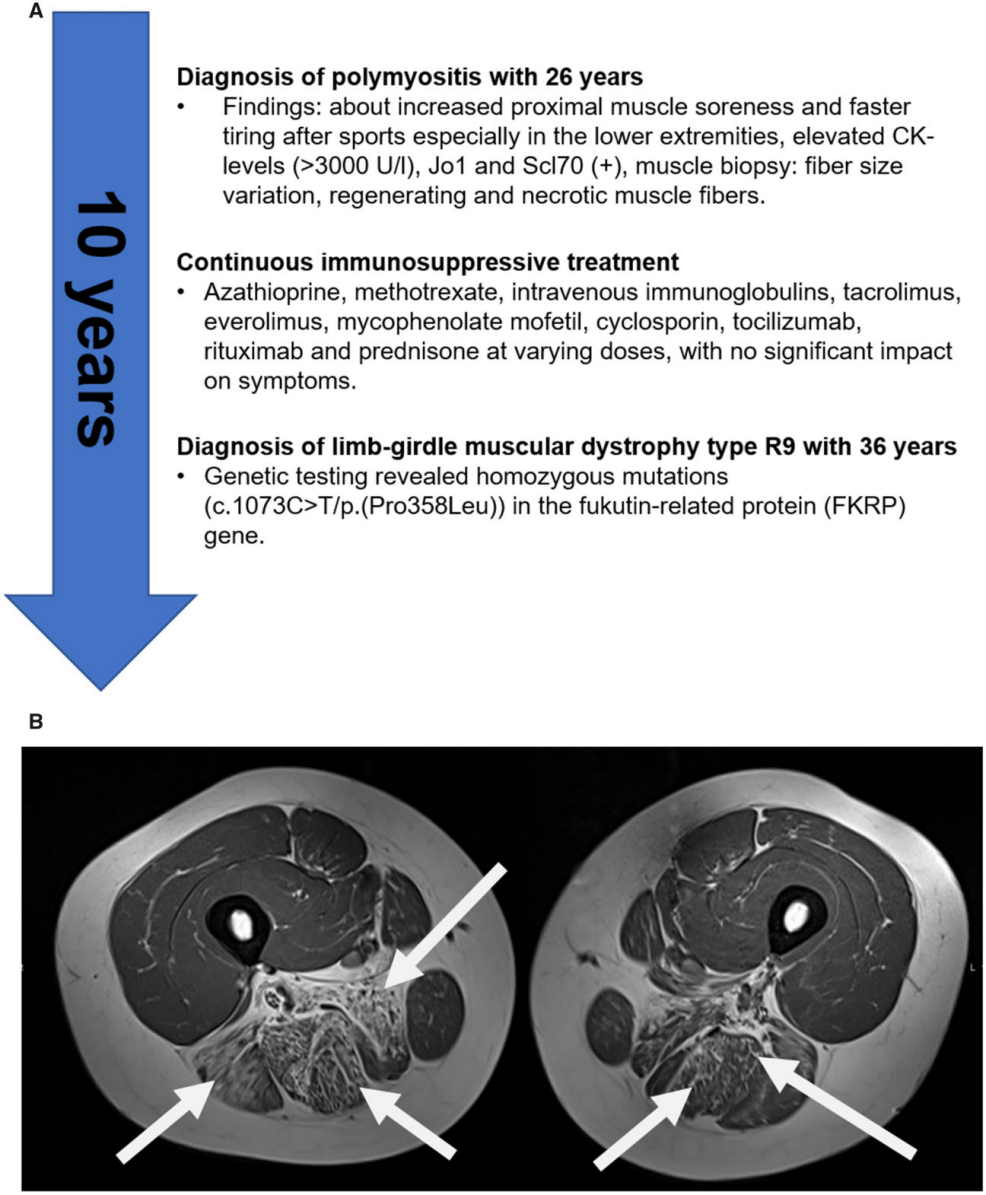
Case overview. (**A**) Case overview. (**B**) T1-weighted MRI of thighs, showing marked fatty atrophy, in particular of biceps femoris (long head), semitendinosus and adductor magnus and longus muscles, typical for limb-girdle muscular dystrophy type R9

With the clinical presentation and course, which were notably refractory to immunosuppressive therapy, in addition to the laboratory, radiological, neurophysiological and myopathological findings, limb-girdle muscular dystrophy (LGMD) was suspected. Genetic testing revealed homozygous mutations [c.1073C>T/p.(Pro358Leu)] in the fukutin-related protein (FKRP) gene. This mutation has been described as homozygous or compound heterozygous in patients with autosomal-recessive LGMD type R9 (formerly type 2I) [1]. FKRP is involved in the glycosylation of alpha-dystroglycan, which in turn is essential for interaction with the extracellular matrix [2]. LGMD R9 is typically associated with very high CK levels, exertional pain, calf hypertrophy, slowly progressive proximal tetraparesis, dilated cardiomyopathy and/or respiratory insufficiency [2].

This case highlights the risk of misdiagnosing polymyositis/idiopathic inflammatory myopathy in patients with muscular dystrophy. For the patient, the diagnosis was a relief and a shock at the same time. She felt relieved that she no longer needed immunosuppressants but was also anxious about the genetic risk because she was now trying to become pregnant. Intensive psychotherapy was necessary; however, fortunately no relevant muscle weakness progression occurred.

Signs to look for are therefore: asymptomatic high CK levels; slowly progressive course of weakness, especially when refractory to existing immunosuppressive therapy; muscular dystrophy typical pattern of weakness; muscular dystrophy-associated features, such as calf hypertrophy, absent myositis-specific or associated autoantibodies and predominant fatty atrophy; and a typical muscular dystrophy pattern in muscle MRI [3, 4]. It should be noted that very high CK levels, negative family history, (weak) positive myositis-associated or specific antibodies, (unspecific) inflammatory changes in muscle biopsy [5] and muscle oedema on MRI [6] can also be seen in patients with muscular dystrophies.

In conclusion, thorough multidisciplinary work-up and synopsis of all clinical results are important before diagnosing idiopathic inflammatory myopathy. This will help to avoid potentially harmful immunosuppressive therapies and allow the best care of patients with non-inflammatory myopathies, including regular neuromuscular, cardiac and pulmonary check-ups [4]. Furthermore, an atypical clinical presentation or course in patients with an existing diagnosis of idiopathic inflammatory myopathy should prompt a re-evaluation of the diagnosis.

## Data Availability

The data supporting the conclusions of this article will be made available by the authors, without undue reservation.

## References

[1] Boito CA , Fanin M, Gavassini BF et al Biochemical and ultrastructural evidence of endoplasmic reticulum stress in LGMD2I. Virchows Arch 2007;451:1047–55. 17952692 10.1007/s00428-007-0515-3

[2] Richard I , Laurent JP, Cirak S et al; ENMC FKRP Study Group. 216th ENMC international workshop: clinical readiness in FKRP related myopathies January 15–17, 2016 Naarden, The Netherlands. Neuromuscul Disord 2016;26:717–24. 27639504 10.1016/j.nmd.2016.08.012

[3] Straub V , Carlier PG, Mercuri E. TREAT-NMD workshop: pattern recognition in genetic muscle diseases using muscle MRI: 25–26 February 2011, Rome, Italy. Neuromuscul Disord 2012;22(Suppl 2):S42–53. 22980768 10.1016/j.nmd.2012.08.002

[4] Marago I , Roberts M, Roncaroli F et al Limb girdle muscular dystrophy R12 (LGMD 2L, anoctaminopathy) mimicking idiopathic inflammatory myopathy: key points to prevent misdiagnosis. Rheumatology (Oxford) 2022;61:1645–50. 34264321 10.1093/rheumatology/keab553

[5] Tidball JG , Welc SS, Wehling-Henricks M. Immunobiology of inherited muscular dystrophies. Compr Physiol 2018;8:1313–56 30215857 10.1002/cphy.c170052 PMC7769418

[6] Lehmann Urban D , Mohamed M, Ludolph AC, Kassubek J, Rosenbohm A. The value of qualitative muscle MRI in the diagnostic procedures of myopathies: a biopsy-controlled study in 191 patients. Ther Adv Neurol Disord 2021;14:1756286420985256. 33737953 10.1177/1756286420985256 PMC7934066

